# Understanding the Impacts of Soil, Climate, and Farming Practices on Soil Organic Carbon Sequestration: A Simulation Study in Australia

**DOI:** 10.3389/fpls.2016.00661

**Published:** 2016-05-18

**Authors:** Cécile M. Godde, Peter J. Thorburn, Jody S. Biggs, Elizabeth A. Meier

**Affiliations:** Agriculture Flagship, Commonwealth Scientific and Industrial Research Organisation, St. LuciaQLD, Australia

**Keywords:** agricultural practices, APSIM model, climate, conservation practices, crop management, greenhouse gases, nitrous oxide emissions, soil organic matter

## Abstract

Carbon sequestration in agricultural soils has the capacity to mitigate greenhouse gas emissions, as well as to improve soil biological, physical, and chemical properties. The review of literature pertaining to soil organic carbon (SOC) dynamics within Australian grain farming systems does not enable us to conclude on the best farming practices to increase or maintain SOC for a specific combination of soil and climate. This study aimed to further explore the complex interactions of soil, climate, and farming practices on SOC. We undertook a modeling study with the Agricultural Production Systems sIMulator modeling framework, by combining contrasting Australian soils, climates, and farming practices (crop rotations, and management within rotations, such as fertilization, tillage, and residue management) in a factorial design. This design resulted in the transposition of contrasting soils and climates in our simulations, giving soil–climate combinations that do not occur in the study area to help provide insights into the importance of the climate constraints on SOC. We statistically analyzed the model’s outputs to determinate the relative contributions of soil parameters, climate, and farming practices on SOC. The initial SOC content had the largest impact on the value of SOC, followed by the climate and the fertilization practices. These factors explained 66, 18, and 15% of SOC variations, respectively, after 80 years of constant farming practices in the simulation. Tillage and stubble management had the lowest impacts on SOC. This study highlighted the possible negative impact on SOC of a chickpea phase in a wheat–chickpea rotation and the potential positive impact of a cover crop in a sub-tropical climate (QLD, Australia) on SOC. It also showed the complexities in managing to achieve increased SOC, while simultaneously aiming to minimize nitrous oxide (N_2_O) emissions and nitrate leaching in farming systems. The transposition of contrasting soils and climates in our simulations revealed the importance of the climate constraints on SOC.

## Introduction

Soils can act as a net source or sink of atmospheric carbon dioxide (CO_2_) and thus influence the process of global climate change. Their capacity to sequester carbon (C) is huge, as world soils constitute the largest terrestrial reserve of C, sequestering over 2400 Gt (billion metric tons) to a depth of 2 m, more than four times the amount of C in terrestrial biota and three times that in the atmosphere (Hillel and Rosenzweig, 2011). In Australia, agriculture is estimated to contribute to 16% of all greenhouse gas (GHG) emissions (Commonwealth of Australia, 2014). Cosier et al. (2009) explained that if Australia were to capture just 15% of the biophysical capacity of Australian soils and vegetation to store C, it would offset the equivalent of 25% of Australia’s current annual greenhouse emissions for the next 40 years (15% of 1,017 million tons (Mt) = 153 Mt).

Furthermore, increasing soil organic carbon (SOC) is widely regarded as beneficial to soil fertility, soil structure, nutrient retention, water holding capacity, reduced soil erosion, and is, therefore, integral to sustainable farming (Sanderman et al., 2010; Hoyle, 2013). These improvements in soil properties are of high importance in Australia where the soils are ancient and have intrinsically low levels of organic matter in their surface layers. For instance, the average stock of SOC in the 0–0.3 m layer is estimated to be 29.7 t/ha in Australia (Viscarra Rossel et al., 2014), which is half that in France (59.9 t/ha; Martin et al., 2011) and about two thirds that in Brazil (about 44 t/ha; Batjes, 2005).

Grain cropping constitutes a major component of the Australian agricultural industry, with approximately 22 million hectares sown to grains in 2009–2010 (Pricewaterhouse Coopers, 2011). SOC in these farmlands is strongly influenced by human activities. For example, in a meta-analysis, Luo et al. (2010) showed that SOC in the surface 0.1 m of Australian cultivated soils was 51% lower than in adjacent natural ecosystems.

Unfortunately, as in many parts of the world (Luo et al., 2010), the review of literature pertaining to SOC dynamics within Australian grain farming systems does not enable us to provide advice on farming practices that will unerringly increase or maintain SOC for a specific combination of Australian soil and climate. Indeed, although in general, the adoption of conservation farming practices (zero tillage, stubble retention, and crop rotation) increased SOC and improved soil physical and chemical properties, some studies found that it is not always the case: Examples include studies on tillage (Gupta et al., 1994; Fettell and Gill, 1995; Armstrong et al., 2003; Dalal et al., 2007; Luo et al., 2010); and studies on stubble management (Gupta et al., 1994; Fettell and Gill, 1995; Valzano et al., 2001; Luo et al., 2010). Furthermore, most of the studies were based on a limited number of experiments conducted at specific locations, for short periods, and where the soil was sampled to a shallow depth (e.g., 0–0.15 m), making it even harder to make solid conclusions on the practices beneficial to SOC (Luo et al., 2014).

**FIGURE 1:**
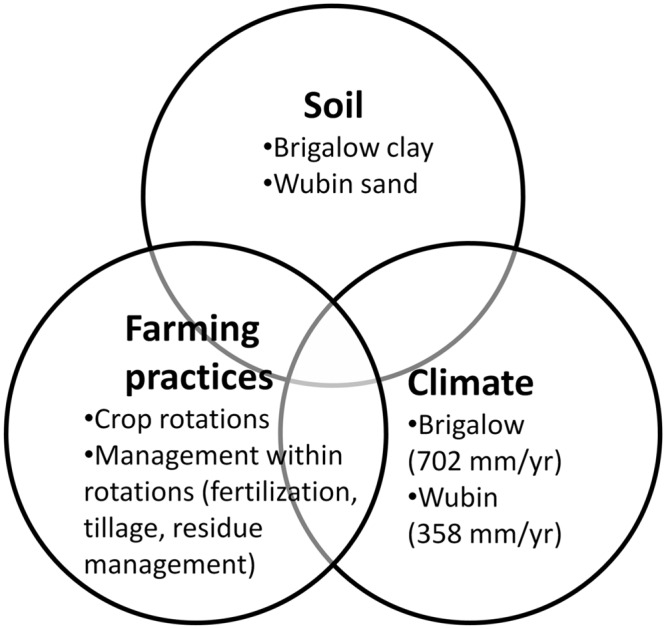
**The study analyzed the influences of the interactions of soil, climate, and farming practices on **soil organic carbon** (SOC) by undertaking a factorial analysis of contrasting soils, contrasting climates and farming practices**.

In this paper, we examined the complex interactions of soil, climate, and farming practices on SOC through a more systematic approach with the Agricultural Production Systems sIMulator (APSIM) agro-ecological model. By transposing contrasting soils and climates in this Australian case-study (**Figure 1**), we aimed to think beyond the boundary of existing soil and climate patterns.

## Materials and Methods

### APSIM: A Modeling Framework

The modeling framework used in this study was APSIM version 7.6 (Holzworth et al., 2014). APSIM is an agro-ecological model that has been more extensively validated in simulating long-term SOC dynamics in Australian crop-lands than other models, and has been used in a wide range of studies dealing with crop rotation, tillage, stubble, and fertilization managements (Huth et al., 2010; Thorburn et al., 2010; Luo et al., 2011; Zhao et al., 2013).

### Modeling Approach

**FIGURE 2:**
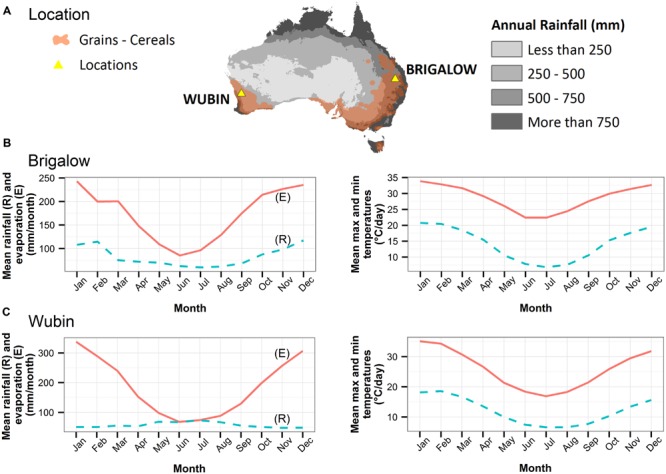
**Locations of Wubin, Brigalow, grain production regions, and rainfall areas (A).** Average monthly values for rainfall (R), pan evaporation (E), maximum daily temperature and minimum daily temperature for Brigalow **(B)** and Wubin **(C)** (Jeffrey et al., 2001). National climate data from Australian Government - Bureau of Meteorology (n.d.).

We simulated a factorial combination of contrasting levels of soil, climate, and farming practices found in the Australian grains production region to study their influences on SOC (**Table 1**). For example, (*soil*) Brigalow Vertosol under (*climate*) Wubin climate, with (*farming practices*) a fertilization rate of 50 kg N/ha/year, a wheat–wheat rotation, zero tillage, and stubble burning, was one of the 144 soil–climate–farming practices combinations simulated. The two soils (clay and sandy soils) and climates (702 and 358 mm/year of rainfall) selected are contrasting for the Australian grain cropping region (**Figure 2**). Indeed, the grain-cropping region is characterized by clay soils in the east and sandy soils in the west and by subtropical and tropical climates in the north and Mediterranean climates in the south. Although the crop rotations and farming practices were realistic for Australia, the modeling was a sensitivity-type data analysis and hence was not representing actual experiments and did not aim to represent any specific farming system at a particular location. Furthermore, the modeling did not aim to describe all soil textures and climates in Australian grains lands. The simulations lasted 90 years (from January 1, 1924 to December 31, 2013).

**Table 1:** Explanatory variables of the Agricultural Production Systems sIMulator model and their levels.

Variable to explain	Explanatory variables	Contrasting levels
Total soil organic carbon (SOC) in the 0–0.3 m layer	Soil type	Brigalow gray Vertosol [clay, moderate organic C (0–0.3 m): 1.1%]
		Wubin deep yellow sand [sand, low organic C (0–0.3 m): 0.4%]
	Climate type	Brigalow climate (high rainfall: 702 mm/year)
		Wubin climate (low rainfall: 358 mm/year)
	Amount of fertilizer (kg N/ha/year)	0
		50
		100
	Rotation type	Wheat–wheat
		Wheat–chickpea (crop of the 1st year simulated: chickpea)
		Chickpea–wheat (crop of the 1st year simulated: wheat)
	Tillage practice	Zero tillage
		Conventional tillage
	Stubble practice	Stubble retained
		Stubble burnt

*Example of a combination of soil with climate and farming practices, which represents one of the 144 simulations: Brigalow gray Vertosol under Wubin climate, with a fertilization rate of 50 kg N/ha/year, a wheat–wheat rotation, zero tillage, and stubble burning.*

The two contrasting climates in the simulations were Brigalow and Wubin climates. Brigalow climate (QLD, Australia, 26 51′S, 150 47′E) is characterized by high annual rainfall (702 mm/year), with most rainfall (71%) occurring during the warmer months of October to March. In contrast, Wubin climate (WA, Australia, 30 06′S, 116 38′E) is characterized by low annual rainfall (358 mm/year) with most rainfall (63%) occurring during the winter months (May–July; **Figure 2**). Rainfall at Wubin is very low during the summer months. The two contrasting soils in the simulations were Brigalow gray Vertosol, a cracking clay soil with a moderate SOC content (1.1% in the 0–0.3 m layer) and Wubin deep yellow sand, with a low SOC content (0.4% in the 0–0.3 m layer; **Table 2**). These climates (**Figure 2**) and soils (Luo et al., 2013) approximately span the range found in Australian grains producing areas.

**Table 2:** Initial SOC in Wubin sand and Brigalow clay (soil data collected by CSIRO).

Layer (m)	SOC (%)	
	Wubin sand	Brigalow clay
0–0.1	0.67	1.19
0.1–0.2	0.29	1.1
0.2–0.3	0.25	1.01
0.3–1.5	0.13	0.28

### Model Configuration

Long-term Brigalow and Wubin climate data originated from the SILO climate database (Queensland Government - Department of Science Information Technology Innovation and the Arts, 2014). APSIM soil data came from measurements made in the soils at the study locations, stored in the APSIM-APSoil database (APSIM Initiative, n.d.).

APSIM is a component-based model (Holzworth et al., 2014). During the simulations, different modules calculated soil and crop processes interacted on a daily time step, driven by climate data and crop management activities. The main processes simulated in this study were C and N dynamics in soil including nitrous oxide (N_2_O) emission (APSIM-SoilN; Probert et al., 1998; Thorburn et al., 2010), soil water dynamics (APSIM-SoilWat; Probert et al., 1998), soil temperature (APSIM-SoilTemp), plant growth, and residue dynamics (APSIM-SurfaceOM; Probert et al., 1998; Thorburn et al., 2001). Management processes such as rotation, tillage, stubble management, sowing, fertilization, and harvest were applied via the APSIM-Manager module to represent the operations conducted during the simulations. The APSIM-wheat and APSIM-chickpea modules simulated the growth and plant development on a daily time-step on an area basis, not for a single plant. Plant growth responded to climate (temperature, rainfall, and radiation), soil water, and nitrogen supply (Keating et al., 2003). The dynamics of water, N, C, and roots were simulated in soil layers, with water [and associated nitrate (${\mathrm{NO}}_{3}^{-}$)] moving between layers where gradients existed. The soil water module used in this study was a ‘cascading bucket’ water balance model. N mineralization, N immobilization, nitrification (following Probert et al., 1998), and denitrification (following Thorburn et al., 2010) were explicitly described in each layer. Soil moisture, pH, and temperature affected all soil N-cycling processes.

N_2_O emissions in APSIM were modeled as originating from nitrification and denitrification (Thorburn et al., 2010). Nitrification in the APSIM-SoilN model followed Michaelis–Menten kinetics and was modified by pH, soil moisture, and temperature (Probert et al., 1998). N_2_O emissions during nitrification were calculated as a fixed proportion of 0.2% of nitrified N. Denitrification was simulated as first-order reaction dependent on ${\mathrm{NO}}_{3}^{-}$. It was further driven by active C, temperature, and soil aeration. Soil aeration was represented by a soil moisture factor increasing from zero to one for moisture contents between drained upper limit (DUL) and saturation (SAT). This assumed that denitrification took place only at water contents above DUL. Denitrification resulted in N_2_ and N_2_O, formed at a ratio which depended on the quotient of ${\mathrm{NO}}_{3}^{-}$ concentration to respired CO_2_ as well as water filled pore space. A detailed description of the nitrification and denitrification processes was given by Probert et al. (1998) and Thorburn et al. (2010).

### Model Testing

**FIGURE 3:**
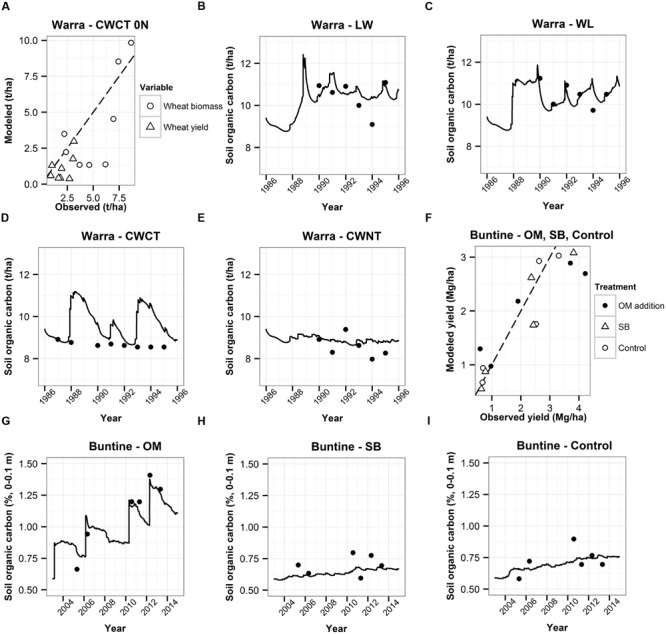
**Modeled and observed plant biomass and yields for Warra **(A)** and modeled and observed yields for Buntine **(F)**.** Modeled (lines) and observed (dots) soil organic carbon (SOC; 0–0.1 m) at Warra **(B–E)** and Buntine **(G–I)** under different agricultural practices over time. For Warra, the different agricultural practices were the following: lucerne–wheat (LW) and wheat–lucerne (WL) rotations and continuous wheat (CW) with conventional tillage (CT), and without tillage (NT). The CWCT treatment represented in **(A)** was not fertilized (After Luo et al., 2011). For Buntine, the different agricultural practices were the following: organic matter (OM) addition, stubble burning (SB), and control (minimum till with knife points and full stubble retention).

We tested the APSIM modeling capability by using the detailed data from Warra (26.93°S, 150.92°E), 16 km southeast of the Brigalow town and from Buntine (30 04′S, 116 13′E), 15 km north of the Wubin town. Data for Warra originated from Dalal et al. (1995) and Luo et al. (2011), and data for Buntine from Liebe Group (2012, 2015). The Warra soil and climate are similar to the Brigalow soil and climate used in the APSIM model described in the previous paragraph. Furthermore, the Buntine soil and climate are similar to the Wubin soil and climate. Configuration of the model was undertaken using a wide range of available information. Agronomic records of sowing dates, cultivar selection, plant populations, tillage, and weed spraying were used to reproduce the historical management. APSIM was able to adequately describe the plant biomass and grain yields in Warra in some cases (**Figure 3A**). In others, the differences between modeled and observed values could be explained by the field experiments not being specifically designed for APSIM testing and thus insufficient information being collected by the experimenters. Furthermore, as the model configuration done by Luo et al. (2011) was based on field experiment descriptions given by Dalal et al. (1995), there might have been some information missing for a model configuration accurately representative of the field experiments. More importantly, despite some discrepancies described above, SOC was well represented as APSIM was able to adequately model changes in soil C content within the surface soil layers for most of the years in Warra and Buntine (**Figure 3**).

We also tested the model by comparing observed and modeled yields and wheat harvest indexes when combining Wubin soil and climate and Brigalow soil and climate. In some cases, simulations of crop productivity were similar to the farmer’s yields in Wubin and Brigalow regions (**Tables 3** and **4**). In others, simulations over-predicted yields, likely due to factors not represented within the APSIM model such as nutrient deficiencies/toxicities (non-N), weeds, pests, and diseases, severe frost or excessive heat (Mason, 1992; Luo et al., 2011).

**Table 3:** Comparison between average yields from the APSIM simulations (1924–2013) combining Wubin soil plus Wubin climate, literature, and surveys developed as part of CSIRO’s project ‘Achieving least cost greenhouse gas (GHG) abatement-opportunities in Australian grains farms’.

	Wheat					Chickpea			
	APSIM simulations: Wubin soil + Wubin climate			Farmers involved in CSIRO’s project^a^—Wubin	Mason (1992) – Woogan Hills, WA / Nabawa, WA	APSIM simulations: Wubin soil + Wubin climate			Farmers involved in CSIRO’s project^a^—Wubin
N-Fertilizer rate (kg N/ha/year)	0	50	100	80	50	0	50	100	80
Average yield (kg/ha/year)	677	2055	2574	1900	1838/2200	1517	1483	1457	900

*^a^Project’s name: Achieving least cost GHG abatement-opportunities in Australian grains farms.*

**Table 4:** Comparison between yields from the APSIM simulations (1924–2013) combining Brigalow soil plus Brigalow climate, literature, and surveys developed as part of CSIRO’s project ‘Achieving least cost GHG abatement-opportunities in Australian grains farms’.

	Wheat							Chickpea			
	APSIM simulations: Brigalow soil + Brigalow climate			Fertilizer Industry Federation of Australia CSIRO (2006)—Dalby, QLD, Australia				APSIM simulations: Brigalow soil + Brigalow climate			Farmers involved in CSIRO’s project^a^—Brigalow
N-Fertilizer rate (kg N/ha/year)	0	50	100	0	40	60	80	0	50	100	70
Average yield (kg/ha/year)	1664	2652	3039	2000	2500	3000	3000	2191	2162	2126	1500

*^a^Project’s name: Achieving least cost GHG abatement-opportunities in Australian grains farms.*

**FIGURE 4:**
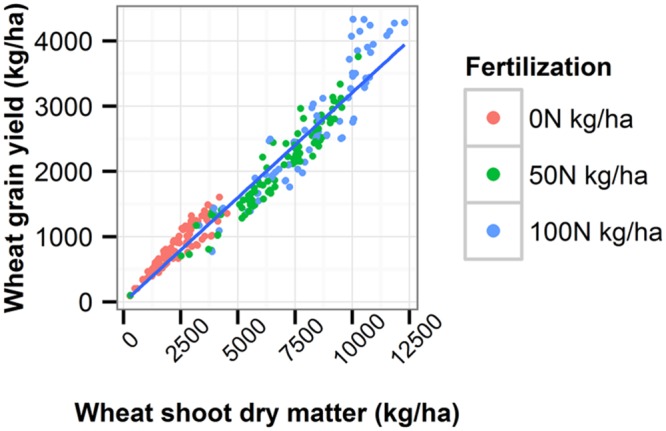
**Relationship between wheat grain yield and wheat shoot dry matter.** The blue line is the linear regression line, the slope of which defines the harvest index across all treatments. Data is from the Agricultural Production Systems sIMulator simulation combining Wubin climate and soil, conventional tillage and stubble burning. Simulated period: 1924–2013.

The harvest index, which is the ratio of harvested grain to total shoot dry matter, was quite well simulated by APSIM compared to the maximum harvest index given by Unkovich et al. (2010)’s review on harvest index for Australian field crops (**Figure 4**; **Table 5**).

**Table 5:** Comparison of harvesting index between APSIM simulations and literature.

Harvest index	Literature dryland crop (Unkovich et al., 2010)	APSIM – Brigalow soil and climate	APSIM – Wubin soil and climate
Chickpea	Mean:0.37; Max: 0.55	Min: 0.51; Max: 0.55	Min: 0.54; Max: 0.63
Wheat	Mean:0.37; Max : 0.56	Min: 0.30; Max: 0.42	Min: 0.30; Max: 0.34

*Simulated period: 1924–2013.*

Furthermore, prediction of SOC after 90 years of production was coherent with literature (Farmers’ data; White, 1990; Luo et al., 2011; Soil Quality Pty Ltd, 2014; The Soil Carbon Research Program - CSIRO Australian Universities and State Government Agencies, 2014).

### Statistical Analyses

We undertook a sensitivity analysis to study how the uncertainty in the model’s output, SOC, can be apportioned to different sources of uncertainty in its inputs called explanatory variables. Here, the explanatory variables were the soil, climate, fertilization, tillage, stubble and rotation managements (**Table 1**) and the statistical model was:

(1) $$E(Y|X_{1},\ldots ,X_{p})=\alpha +\beta_{1}X_{1}+\cdots +\beta_{p}X_{p}+\varepsilon$$

Where *Y* is the response measurement SOC, *X_i_* is the explanatory variable *i* (soil, climate, fertilization, tillage, stubble, and rotation managements), α is the intercept, the β*_j_* are the slopes or coefficients and 𝜀 the errors.

Since we have a linear model, a combination of *R*^2^ (coefficient of determination) of the explanatory variable alone with semi-partial *R*^2^ was an efficient way to summarize the influence of the variables on the SOC. *R*^2^ of the explanatory variable *i* and the semi-partial *R*^2^ represent the contribution of the variable alone and the contribution of the variable with its interaction with other variables to the SOC variance, respectively.

We calculated *R*^2^ of the explanatory variable *X_i_* for the model including only the explanatory variable *i*:

(2) $$E(Y|X_{i})=\alpha +\beta_{i}\times X_{i}+\varepsilon$$

Where *Y* is the response measurement SOC, *X_i_* is the explanatory variable *i* (soil, climate, fertilization, tillage, stubble, and rotation managements), α is the intercept, the β*_i_* is the slope or coefficient and 𝜀 the errors.

We calculated semi-partial *R*^2^ of the explanatory variable *i* by the difference between the *R*^2^ of the general model and the *R*^2^ of the general model without the explanatory variable *i* (and consequently without all the interactions where this variable *i* is involved). Semi-partial *R*^2^ represents the increase of *R*^2^ due to the addition of the variable *i* in the model.

We undertook another statistical analysis of the influence of soil, climate, fertilization, tillage, stubble, and rotation managements on the rate of loss of SOC over the 90 years simulated. We used an analysis of covariance (ANCOVA) to model the relationship between the scalar dependent variable ‘SOC in 0–0.3 m layer’ and the multiple explanatory variables: time, soil, climate, fertilization, tillage, stubble, and rotation managements (Eq. 1).

Because the residuals of the ANCOVA were time dependent (autocorrelation analysis significant), we smoothed short-term fluctuations to highlight longer-term trends. Hence, we calculated the moving average of order 6 for SOC for each combination of soil, climate, fertilization, tillage, stubble, and rotation managements for each year.

Then, we used the test of the interaction between the variable time and the other explanatory variables of the ANCOVA model to compare the slopes of the lines of linear regressions corresponding to the rate of loss of SOC over time for the different variables (soil, climate, fertilization, etc.).

A correlation matrix based on Pearson parametric correlation test investigated the dependence between SOC change over the 90 years simulated and average chickpea yield. The Pearson product–moment correlation coefficient referred as *r* is a measure of the linear correlation between SOC change over the 90 years simulated and average chickpea yield, giving a value between +1 and -1 inclusive, where 1 is total positive correlation, 0 is no correlation, and -1 is total negative correlation.

## Results

We studied the relative contribution of the variables soil, climate, and farming practices to SOC variations for the first and last 10 years simulated, as well as the relative contribution of farming practices when the soil and climate are fixed. We found, for instance, that the last 10 years of the simulations, the type of soil (i.e., Brigalow soil or Wubin soil, **Table 2**) and its interactions with the climate and the farming practices explained 66% of the SOC variation. The type of climate, fertilization rate, rotation type, stubble practice, and tillage and their interactions explained, respectively, 18, 15, 3, 2, and 1% (*R*^2^ = 99.79). We also examined the influences of the variables’ levels (e.g., Wubin soil vs. Brigalow soil, **Table 1**) on SOC over the 90 years simulated, and then investigated more specifically the influence of crop rotation and climate on SOC. We found that when fertilizer was applied, during the last 10 years of the simulations SOC was on average 7.2% higher under a wheat–wheat rotation than under a wheat–chickpea rotation. When no fertilizer was applied, the simulated wheat yields were 67% higher and SOC in the 0–0.3 m layer was 23% higher in a wheat–chickpea rotation compared to a wheat-wheat rotation. Moreover, under Brigalow climate, for 13 of the 16 scenarios, SOC decreased or was maintained over time. Eventually, we identified some potential environmental trade-offs associated with practices that increased SOC.

### Impacts of Soil, Climate, and Farming Practices on SOC

Results of the sensitivity analysis showed that the type of soil (i.e., Brigalow soil or Wubin soil, **Table 2**) and its interactions with the climate and the farming practices explained 96% of the SOC variation during the first 10 years and 66% during the last 10 years. The importance of soil was to be expected, given that there was a large difference in the initial SOC between Brigalow and Wubin soils and initial SOC had a large influence on the trajectory of SOC. The type of climate (i.e., Brigalow climate or Wubin climate) and its interactions with the other explanatory variables also had a significant influence on the variation of SOC (first 10 years: 3%, last 10 years: 18%). Fertilization rate had a smaller influence, and the effect was most strongly pronounced after several decades of constant practices SOC (last 10 years: 15%). Rotation type, stubble practice and tillage practice had much lower levels of influence when compared to the other explanatory variables (last 10 years: respectively, 3, 2, and 1%).

**FIGURE 5:**
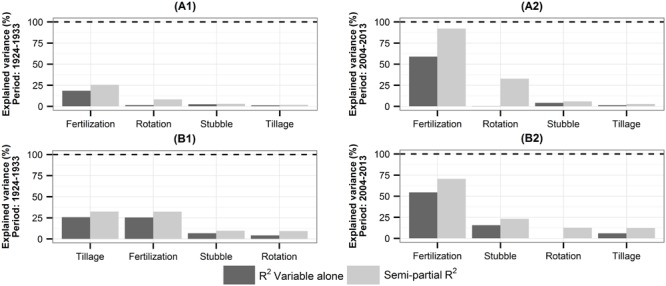
**Contribution of the management practices to SOC variations for Brigalow **(A)** and Wubin **(B)** soils and climates. (A1,B1)** First 10 years of the simulations and **(A2,B2)** last 10 years of the simulations. *R*^2^ of the variable *i* and the semi-partial *R*^2^ represent the contribution of the variable alone and the contribution of the variable with its interaction with other variables to the SOC variance, respectively.

The two locations in the study (i.e., Brigalow soil plus climate and Wubin soil plus climate) were separately subjected to a sensitivity analysis to determine if the sensitivity of SOC to management differed between the locations (**Figure 5**).

For Brigalow, the analysis indicated that during the first 10 years, the fertilization practice and its interactions with the other farming practices explained 25% of the variation of SOC, followed by rotation (8%), stubble (3%), and tillage (2%) practices. During the last 10 years, the fertilization practice and its interactions with the other farming practices explained 92% of the variation of SOC, followed by rotation (33%), stubble (6%), and tillage (3%) practices.

For Wubin, the analysis indicated that during the first 10 years, the tillage practice and its interactions with the other farming practices explained 32% of the variation of SOC, fertilization also explained 32%, followed by stubble (10%), and rotation (9%) practices. During the last 10 years, the fertilization practice and its interactions with the other farming practices explained 70% of the variation of SOC, followed by stubble (23%), rotation (13%), and tillage (12%) practices.

Thus, the contributions of the explanatory variables to SOC variations were different between Brigalow and Wubin locations and between the first and last 10 years of the simulations. However, for both locations, during the last 10 years, fertilization was by far the variable that contributed the most to SOC variations (Brigalow: 92% and Wubin: 70%).

As well as investigating the factors associated with the amount of SOC, we examined the factors associated with the rate of SOC loss over the 90 years simulated. The rate of SOC loss averaged across all farming practices and both climates was faster under Brigalow clay than under Wubin sand (**Table 6**). Moreover, it was faster under Brigalow climate than Wubin climate, and faster under a wheat-wheat rotation than a wheat–chickpea rotation. Application of N fertilizer reduced the rate of SOC loss, with the slowest rate of change occurring in the 100 kg N/ha/year treatment, and the fastest rate of change occurring when no fertilizer was applied. Stubble burning led to a faster rate of SOC loss than stubble retention. In addition to this, zero tillage led to a faster rate of SOC loss than conventional tillage.

**Table 6:** Influence of the types of soils, climates and management practices on the rate of SOC loss over the 90 years simulated (1924–2013).

Variable	Level	Value of the linear regressions’ slopes	Influence of the variable’s level on the slope
Soil	Wubin sand	-3.37e^-06^	–
	Brigalow clay	-5.06e^-06^	– –
Climate	Wubin climate	-1.13e^-06^	–
	Brigalow climate	-3.37e^-06^	– –
Rotation	Wheat-wheat	-5.94e^-06^	– –
	Wheat-chickpea	-3.37e^-06^	–
Fertilization	0 kg N/ha/year	-3.37e^-06^	– –
	50 kg N/ha/year	-7.07e^-07^	–
	100 kg N/ha/year	1.30e^-07^	+
Tillage	Conventional tillage	-3.37e^-06^	–
	Zero tillage	-3.98e^-06^	– –
Stubble	Stubble burnt	-3.37e^-06^	– –
	Stubble retained	-2.35e^-06^	–

*The statistical approach is detailed in Section “Statistical Analyses”. The sign ‘–’ indicates that the variable’s level decreases the slope of the rate of SOC loss over time, ‘– –’ indicates that it decreases the slope more than the other variable’s level and ‘+’ indicates that it increases the slope.*

### Effects of Rotation and Fertilizer Application on SOC

When fertilizer was applied, a wheat-wheat rotation resulted in higher SOC compared to a wheat-chickpea rotation. Indeed, the last 10 years of the simulations, SOC was on average 7.2% higher under a wheat-wheat rotation than under a wheat-chickpea rotation. This was because chickpea in a wheat-chickpea rotation led to 47% less biomass C incorporated into the soil organic matter pool (1250 kg/ha/year) than wheat in a wheat-wheat rotation (2375 kg/ha/year). However, when no fertilizer was applied, the leguminous phase increased wheat yields and SOC because of fixed N added to the soil (on average 50 kg/ha/year for the last 10 years of the simulations). For instance, the last 10 years of the simulations, when no fertilizer was applied, the simulated wheat yields were 67% higher and SOC in the 0–0.3 m layer was 23% higher in a wheat–chickpea rotation compared to a wheat–wheat rotation.

### Climate Impacts on SOC

**FIGURE 6:**
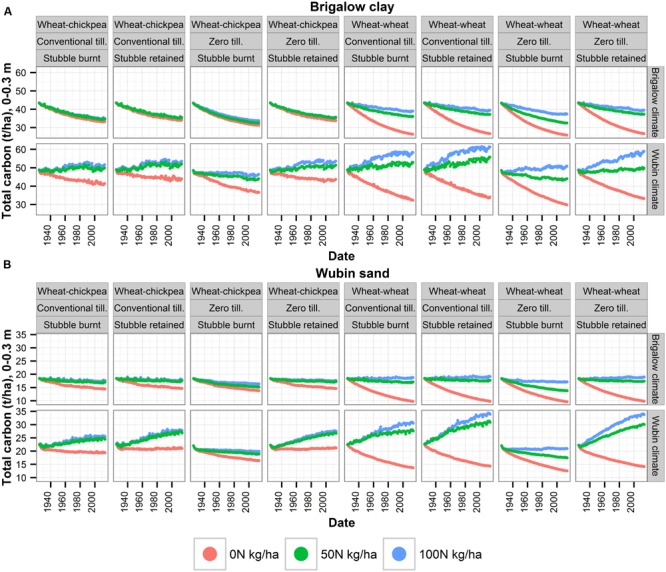
**Influence of soil, climate and different farming practices on SOC (0–0.3 m) over 90 years of APSIM simulations. (A)** Brigalow clay, **(B)** Wubin sand.

Within a particular combination of soil and farming practices, for a given year, SOC was lower under Brigalow climate than under Wubin climate (**Figure 6**). Moreover, under Brigalow climate and for any soil type, SOC always decreased or was maintained over time, except for the three scenarios combining Wubin sand, wheat–wheat, a fertilization rate of 100 kg N/ha/year, and conventional tillage or zero tillage with stubble retention. For these three scenarios, SOC slightly increased over time. On the contrary, under Wubin climate, when fertilizer was applied and for any combination of soil and farming practices, SOC almost always increased. This finding underlined the importance of the climate on SOC.

### Impacts on SOC and the Environment

#### Effects of Yield on SOC

**FIGURE 7:**
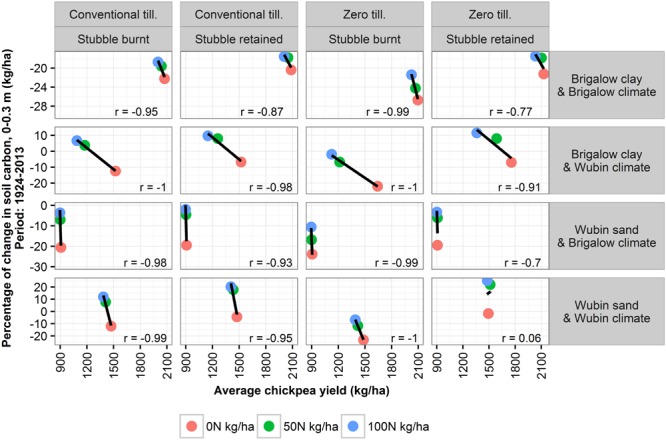
**Correlation between average chickpea yield and percentage of change in SOC (0–0.3 m) over 90 years of APSIM simulations**.

In the simulations, wheat yields increased together with SOC. This correlation was expected because increased simulated wheat yields were the result of higher simulated wheat biomass C, which returned greater quantities of C to the soil through root and residues. In contrast, an increased chickpea yield was often associated with a decrease in SOC (**Figure 7**) suggesting that different mechanisms were controlling the relationship between plant biomass C and soil C in the chickpea simulations. This was due to the higher N concentrations of chickpea residues and roots, making an increased chickpea yield contributing to higher soil N which promoted C mineralization and consequently SOC loss. The potential positive influence of wheat yields on SOC is of interest because it means that the process of increasing SOC is supported by the farmers’ goals to increase wheat yields. However, do the practices that increase wheat yields and, therefore, SOC have only a positive impact on the environment?

#### Effects of Fertilization on SOC and the Environment

**FIGURE 8:**
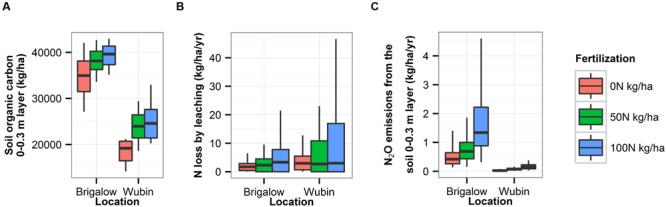
**Influence of the fertilization rate on SOC (0–0.3m) **(A)**, N loss by leaching **(B)** and N_2_O emissions **(C)** for the combination of Brigalow soil plus climate and for the combination of Wubin soil plus climate**.

To increase wheat yields and SOC for a particular combination of soil and climate, the most efficient way was to increase fertilization. However, this led to amplified N leaching and N_2_O emissions, especially in an environment susceptible to leaching like a sandy soil or under a high rainfall climate (**Figure 8**).

#### Effects of Climate and Crop/Fallow Patterns on the Environment

**FIGURE 9:**
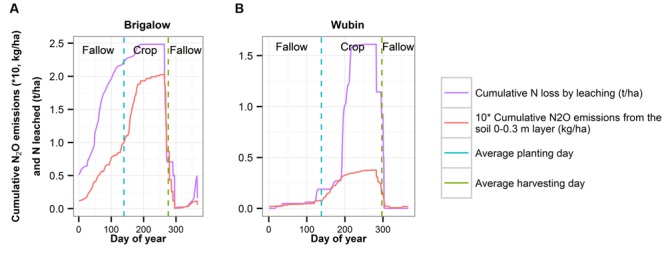
**Simulated cumulative N_2_O emissions and cumulative N loss by leaching during the fallow and the crop periods for Brigalow soil + climate **(A)** and for Wubin soil + climate **(B)**.** Conditions simulated: wheat-wheat rotation with a fertilization rate of 50 kg N/ha/year, average of the last 10 years of the simulations.

When comparing the N leaching process during the fallow period and during the crop period (**Figure 9**), under Brigalow climate, N leaching occurred mainly during the fallow period: during the crop period, plant N uptake effectively reduced the amount of soil N available to be leached. Under Wubin climate, N leaching occurred during crop period because seasonal rainfall during the crop period is higher than in the Brigalow climate, which increased drainage even if plant uptake was present.

When comparing the N_2_O emitted during the fallow period and during the crop period (**Figure 9**), under Brigalow climate, N_2_O emissions were the same during the fallow and crop period, whereas under Wubin climate, N_2_O emissions mainly occurred during the crop period. This difference could be explained because N_2_O emissions were dependent on water availability. Brigalow had higher rainfall, quite homogeneously distributed over the year. Wubin rainfall occurred mainly during the crop period, promoting N_2_O emissions during this period.

## Discussion

### Impacts of Soil, Climate, Management Practices, and Their Interactions on SOC

In this study, SOC was impacted differently by the different combinations of soils, climates and farming practices and can be managed to some degree via simple changes in agronomic practice. This is consistent with the findings of Sperow et al. (2003), Yan et al. (2007), Luo et al. (2010), Zhao et al. (2013), and Robertson et al. (2015). For Brigalow and Wubin sites, fertilization rate explained a much larger proportion of the variability in SOC than rotation, tillage, and stubble management practices during the last 10 years simulated (92 and 70%, respectively), underlining the importance of fertilization for C sequestration. However, simulated results for Brigalow and Wubin sites suggested that increasing the fertilization rate to promote C sequestration can have consequences on the environment by increasing the chance of N loss by leaching and denitrification (with associated N_2_O emissions), especially during the rainy season of tropical and sub-tropical climates and during the fallow period. This trade-off is important as N_2_O is 296 times more potent than CO_2_ as a GHG (Kong et al., 2010) and highlights the complexities in managing SOC, N_2_O emissions, and ${\mathrm{NO}}_{3}^{-}$ leaching together in farming systems. Agricultural practices that truly mitigate climate change cannot simply sequester SOC, but must at the same time limit emissions of other GHGs. Tillage and stubble managements had a limited influence on SOC variations when compared to the influences of soil, climate and other farming practices. Several field studies for Australia did not find a significant positive influence of zero tillage and stubble retention on SOC: that is studies on tillage (Gupta et al., 1994; Fettell and Gill, 1995; Armstrong et al., 2003; Dalal et al., 2007) and studies on stubble management (Gupta et al., 1994; Fettell and Gill, 1995; Valzano et al., 2001). These field studies comprised a factorial of two types of farming practices at a specific site; the influences of soil, climate, and other management practices were not part of that factorial. Therefore, this paper contributes to the reconsideration for Australia of the widespread view that reduced tillage and stubble retention lead to substantial C sequestration in arable soils, a view that has been risen by studies from various parts of the world including in North America (Beare et al., 1994; Dick et al., 1998; Yang and Kay, 2001), Brazil (Sá et al., 2001), Europe (Smith et al., 1998, 2000) and Australia (Standley et al., 1990; Cavanagh et al., 1991; Carter and Mele, 1992; Smettem et al., 1992; Dalal et al., 1995; Heenan et al., 1995; Chan and Hulugalle, 1999; Hulugalle and Entwistle, 1997; Pankhurst et al., 2002).

### Impacts of Crop Rotations with a Leguminous Phase on SOC

The link between fertilizer effects and legume effects on SOC is indirect and complex. N additions, whether from fertilizer application or N returned in residues of N-fixing legume, increase yields (which can drive increased SOC), and increase C mineralization (which can drive decreased SOC). Thus, the net results of increased N additions will be a trade-off between these two processes. This trade-off is further complicated by the fact that increasing the yield of a legume such as chickpea also increases the N returned to the soil. Consequently, changes to SOC rely on the benefit ratio of legume biomass to N returned to the soil. This is why when no fertilizer was added, the inclusion of a legume benefited SOC; the reduction in yield for the year going from wheat to legume was offset by the increase in subsequent wheat yields due to the N fixed from the atmosphere by the legume. When fertilizer was applied, including a leguminous phase in a crop rotation was not a good solution to increase SOC, as the leguminous phase can lead to less incorporated biomass C into the soil organic matter pool than other crops, reducing subsequent SOC. It can also contribute to higher soil N which promotes C mineralization and consequently SOC loss. These findings are supported by the field experiments of Dalal et al. (1995), who found no positive impact of the chickpea phase on SOC. However, the findings are contrary to those of Hoyle et al. (2011), who described the inclusion of green manure as having a positive impact on SOC.

### Climate Impacts on SOC

Under the sub-tropical Brigalow climate and for any soil type, SOC always decreased or was maintained over time, except for the three scenarios combining Wubin sand, wheat–wheat, a fertilization rate of 100 kg N/ha/year, and conventional tillage or zero tillage with stubble retention. For these three scenarios, SOC slightly increased over time (**Figure 6**). On the contrary, under the Mediterranean Wubin climate, SOC increased most of the time when N was added whether the soil was high in SOC (Brigalow) or low (Wubin soil). Consequently, the simulated results of the factorial analysis that combined the two contrasting soils and contrasting climates suggests that climate exerted a greater influence on trends in SOC at these two sites than initial SOC, which is often believed to be the driving factor. Thus, our study complements Zhao et al.’s (2013) finding of the importance of initial SOC. They assessed through modeling changes in SOC over time under different farming practices for three zones with homogeneous soil types and climate attributes. They found that the higher the initial SOC is, the greater the decline in SOC. Our factorial approach allowed us to analyze beyond the existing soil–climate combinations and showed that the pattern described by Zhao breaks down when the Brigalow soil is combined with the Wubin climate. We can therefore conclude that Zhao’s finding was dependent on the local climatic conditions and that initial SOC exerts a great influence on trends in SOC only if the climate is constant. The importance of the climate constraint on SOC underlined in this Australian case-study is supported by Dalal and Mayer (1986) who found that mean annual rainfall largely determined SOC in the six Australian soils sampled in southern Queensland but also by Lal (2004), Yan et al. (2007), and Luo et al. (2010) who carried out research on other countries. In APSIM, SOC sequestration decreases with both temperature and rainfall as SOC decomposition increases with soil water content and high soil temperature (Holzworth et al., 2014). However, high temperature and rainfall can also increase SOC sequestration under certain conditions by enhancing plant growth and therefore biomass returned to the soil. Consequently, the influence of climate on SOC, as shown in this study, depends on other variables such as soil and farming practices. For instance, Davidson and Janssens (2006) indicated that the inherently diverse nature of SOC and environmental constraints obscured the responses of SOC dynamics to warmer temperatures. The study highlights the benefits of considering together soil, climate and farming practices when studying SOC dynamics and can therefore help design future targeted investigations that broaden our knowledge on SOC interactions with the agro-ecosystems and the impacts of climate change on SOC. Indeed, global warming, changes in rainfall and increased atmospheric CO_2_ influence both crop productivity and SOC decomposition and further research need to be done on this topic. For instance, the degree to which temperature influences SOC is still misunderstood (Giardina and Ryan, 2000; Fang et al., 2005; Davidson and Janssens, 2006).

### Cover Crops Impacts on SOC and the Environment

As the Brigalow summer fallow period is wet and hot, there is a higher C mineralization during that period, compared to the drier summer at Wubin. Moreover, as no crop is planted, the C inputs into the soil are limited. Consequently, the fallow period at Brigalow is subject to SOC decrease, which can have a negative impact in a short and long term on the soil biological, physical and chemical properties (Peverill et al., 1999; Hoyle, 2013). Growing a cover crop in summer in Brigalow, and in other locations that experience enough rainfall during the usual fallow period could increase SOC in two ways: (i) it would increase the C biomass inputs and (ii) keep the soil drier by plant water uptake, and hence decrease C mineralization. Cover crops in Wubin and to a larger extent in semi-arid climates are not likely to have these SOC benefits because of a lack of water for crop growth during the fallow. The use of cover crops is encouraged in several publications (Sanderman et al., 2010; Hoyle et al., 2011; Thorburn et al., 2013), but its potential under tropical and sub-tropical climates might have often been underestimated in the literature. There is an additional benefit of growing a cover crop in summer under Brigalow and regions with similar rainfall patterns. Indeed, the fallow period was the most susceptible to N leaching (**Figure 9**) and cover crops, by absorbing the excess of N, and taking up soil water, can be a solution to limit N in the underground water and N returned to the atmosphere. However, cover crops could lead to insufficient soil water content for the following crop. In addition to this, by covering the soil surface, they could also limit soil water evaporation and consequently could promote N_2_O emissions. Studying the influence of cover crops on soil water content and examining which of the limited soil water evaporation and water uptake during cover cropping has a stronger effect on soil water content and on N_2_O emissions could be topics of future modeling research.

## Conclusion

Through APSIM simulations combining contrasting soils, climates, and farming practices, we showed that the initial soil C content had the largest impact on SOC over years, followed by the climate and fertilization practices. Tillage and stubble managements had a lower impact compared to the soil, climate, and other farming practices. Furthermore, the inclusion of a chickpea phase in a wheat-chickpea rotation had a negative impact on SOC when fertilizer was applied. By going beyond the boundary of existing soil and climate patterns, the transposition of contrasting soils and climates revealed the importance of the climate constraints on SOC. The study raised the question of cover crops potential in Brigalow, and other regions with sufficient rainfall during the fallow period, to increase SOC as well as to limit N_2_O emissions. Moreover, we showed some complexities in managing SOC, N_2_O emissions, and ${\mathrm{NO}}_{3}^{-}$ leaching together in farming systems. Agricultural practices that truly mitigate climate change cannot simply sequester SOC, but must at the same time limit emissions of other GHGs. The impact of climate on the SOC balance requires further investigation considering the importance of the climate influence highlighted in this study and the actual context of climate change. Moreover, further research on crop cover use potential in tropical and sub-tropical climates and on concomitant management of SOC and N_2_O emissions in agro-ecosystems are needed.

## Author Contributions

CMG: Conceived and designed the study, performed the experiments, analyzed the data and wrote the paper. PJT: Contributed to the design, analyses and paper redaction. JSB: Contributed to performing some experiments and analyses and EAM: Contributed to performing some experiments.

## Conflict of Interest Statement

The authors declare that the research was conducted in the absence of any commercial or financial relationships that could be construed as a potential conflict of interest.

## References

[B1] APSIM Initiative (n.d.). *APSoil [WWW Document].* Available at: https://www.apsim.info/Products/APSoil.aspx [accessed on November 16, 2015].

[B2] Armstrong R. D. Millar G. Halpin N. V. Reid D. J. Standley J. (2003). Using zero tillage, fertilisers and legume rotations to maintain productivity and soil fertility in opportunity cropping systems on a shallow Vertosol. *Aust. J. Exp. Agric.* 43:141 10.1071/EA01175

[B3] Australian Government - Bureau of Meteorology (n.d.). *Bureau of Meteorology [WWW Document].* Available at: http://www.bom.gov.au [accessed on May 28, 2014].

[B4] Batjes N. H. (2005). Organic carbon stocks in the soils of Brazil. *Soil Use Manag.* 21 22–24. 10.1079/SUM2005286

[B5] Beare M. H. Hendrix P. F. Cabrera M. L. Coleman D. C. (1994). Aggregate-protected and unprotected organic matter pools in conventional- and no-tillage soils. *Soil Sci. Soc. Am. J.* 58 787–795. 10.2136/sssaj1994.03615995005800030021x

[B6] Carter M. R. Mele P. M. (1992). Changes in microbial biomass and structural stability at the surface of a duplex soil under direct drilling and stubble retention in north-eastern victoria. *Aust. J. Soil Res.* 30 493–503. 10.1071/SR9920505

[B7] Cavanagh P. P. Koppi A. J. Mcbratney A. B. (1991). The effects of minimum cultivation after three years on some physical and chemical properties of a red-brown earth at forbes. *N. S. W. Aust. J. Soil Res.* 29 263–270. 10.1071/SR9910263c

[B8] Chan K. Y. Hulugalle N. R. (1999). Changes in some soil properties due to tillage practices in rainfed hardsetting Alfisols and irrigated Vertisols of eastern Australia. *Soil Tillage Res.* 53 49–57. 10.1016/S0167-1987(99)00076-8

[B9] Commonwealth of Australia (2014). Quarterly Update of Australia’s National Greenhouse Gas Inventory. Available at: https://www.environment.gov.au/system/files/resources/7d5f76fe-3128-44dd-bef1-f6fa008f686f/files/nggi-quarterly-update-mar-2014_0.pdf (accessed May 10, 2016).

[B10] Cosier P. Flannery T. Harding R. Karoly D. Lindenmayer D. Possingham F. A. A. (2009). *Optimising Carbon in the Australian Landscape.* Boston: Wentworth Group.

[B11] Dalal R. C. Mayer R. J. (1986). Long-term trends in fertility of soils under continuous cultivation and cereal cropping in southern queensland. ii total organic carbon and its rate of loss from the soil profile. *Aust. J. Soil Res.* 24 281–292. 10.1071/SR9860265

[B12] Dalal R. C. Strong W. M. Cooper J. E. King A. J. (2007). No-tillage and nitrogen application affects the decomposition of 15 N-labelled wheat straw and the levels of mineral nitrogen and organic carbon in a Vertisol. *Aust. J. Exp. Agric.* 47 862–868. 10.1071/EA06118

[B13] Dalal R. C. Strong W. M. Weston E. J. Cooper J. E. Lehane K. J. King A. J. (1995). Sustaining productivity of a Vertisol at Warra, Queensland, with fertilisers, no-tillage, or legumes 1. Organic matter status. *Aust. J. Exp. Agric.* 35 903–913. 10.1071/EA9950903

[B14] Davidson E. A. Janssens I. A. (2006). Temperature sensitivity of soil carbon decomposition and feedbacks to climate change. *Nature* 440 165–173. 10.1038/nature04514 16525463

[B15] Dick W. A. Blevins R. L. Frye W. W. Peters S. E. Christenson D. R. Pierce F. J. (1998). Impacts of agricultural management practices on C sequestration in forest-derived soils of the eastern Corn Belt. *Soil Tillage Res.* 47 235–244. 10.1016/S0167-1987(98)00112-3

[B16] Fang C. Smith P. Moncrieff J. B. Smith J. U. (2005). Similar response of labile and resistant soil organic matter pools to changes in temperature. *Nature* 433 57–59. 10.1038/nature03138 15635408

[B17] Fertilizer Industry Federation of Australia CSIRO (eds). (2006). “Fertilize for profits,” in *Australian Soil Fertility Manual* CSIRO Publishing Melbourne.

[B18] Fettell N. Gill H. (1995). Long-term effects of tillage, stubble, and nitrogen management on properties of a red-brown earth. *Aust. J. Exp. Agric.* 35 923–928. 10.1071/EA9950923

[B19] Giardina C. P. Ryan M. G. (2000). Evidence that decomposition rates of organic carbon in mineral soil do not vary with temperature. *Nature* 404 858–861. 10.1038/35009076 10786789

[B20] Gupta V. V. S. R. Roper M. M. Kirkegaard J. A. Angus J. F. (1994). Changes in microbial biomass and organic matter levels during the first year of modified tillage and stubble management practices on a red earth. *Aust. J. Soil Res.* 32 1339–1354. 10.1071/SR9941339

[B21] Heenan D. McGhie W. Thomson F. Chan K. (1995). Decline in soil organic carbon and total nitrogen in relation to tillage, stubble management, and rotation. *Aust. J. Exp. Agric.* 35 877–884. 10.1071/EA9950877

[B22] Hillel D. Rosenzweig C. (2011). “The role of soils in climate change,” in *Handbook of Climate Change and Agroecosystems, Impacts, Adaptation, and Mitigation* eds Hillel D. Rosenzweig C. (London: Imperial College Press) 9–20.

[B23] Holzworth D. P. Huth N. I. Peter G. Zurcher E. J. Herrmann N. I. Mclean G. (2014). APSIM - Evolution towards a new generation of agricultural systems simulation. *Environ. Model. Softw.* 62 327–350. 10.1016/j.envsoft.2014.07.009

[B24] Hoyle F. (2013). *Managing Soil Organic Matter: A Practical Guide.* Kingston, ACT: Central Queensland Soil Health.

[B25] Hoyle F. C. Baldock J. A. Murphy D. V. (2011). “Soil organic carbon – role in rainfed farming systems,” in *Rainfed Farming Systems* eds Tow P. Cooper I. Partridge I. Birch C. (Dordrecht: Springer) 339–361. 10.1007/978-1-4020-9132-2

[B26] Hulugalle N. R. Entwistle P. (1997). Soil properties, nutrient uptake and crop growth in an irrigated Vertisol after nine years of minimum tillage. *Soil Tillage Res.* 42 15–32. 10.1016/S0167-1987(96)01104-X

[B27] Huth N. I. Thorburn P. J. Radford B. J. Thornton C. M. (2010). Impacts of fertilisers and legumes on N_2_O and CO_2_ emissions from soils in subtropical agricultural systems: a simulation study. *Agric. Ecosyst. Environ.* 136 351–357. 10.1016/j.agee.2009.12.016

[B28] Jeffrey S. J. Carter J. O. Moodie K. B. Beswick A. R. (2001). Using spatial interpolation to construct a comprehensive archive of Australian climate data. *Environ. Model. Softw.* 16 309–330. 10.1016/S1364-8152(01)00008-1

[B29] Keating B. A. Carberry P. S. Hammer G. L. Probert M. E. Robertson M. J. Holzworth D. (2003). An overview of APSIM, a model designed for farming systems simulation. *Eur. J. Agron.* 18 267–288. 10.1016/S1161-0301(02)00108-9

[B30] Kong A. Y. Y. Gentile R. Chivenge P. Fonte S. J. Six J. (eds) (2010). “Trade-offs Associated with Using Soil Carbon Sequestration as Climate Change Mitigation,” in *Handbook of Climate Change And Agroecosystems, Impacts, Adaptation, and Mitigation.* London: Imperial College Press 365–392. 10.1142/9781848166561_0019

[B31] Lal R. (2004). Soil carbon sequestration in India. *Clim. Change* 65 277–296. 10.1023/B:CLIM.0000038202.46720.37

[B32] Liebe Group (2012). *Trial Reports [WWW Document].* Available at: http://www.liebegroup.org.au/publications/ [accessed on December 15, 2015].

[B33] Liebe Group The Liebe Group (2015). *Long Term Research Site [WWW Document].* Available at: http://www.liebegroup.org.au/trial-programs-3/liebe-group-long-term-research-site-2010/ [accessed on September 6, 2015].

[B34] Luo Z. Wang E. Baldock J. Xing H. (2014). Potential soil organic carbon stock and its uncertainty under various cropping systems in Australian cropland. *Soil Res.* 52 463–475. 10.1071/SR13294

[B35] Luo Z. Wang E. Bryan B. A. King D. Zhao G. Pan X. (2013). Meta-modeling soil organic carbon sequestration potential and its application at regional scale. *Ecol. Appl.* 23 408–420. 10.1890/12-0672.1 23634591

[B36] Luo Z. Wang E. Sun O. J. (2010). Soil carbon change and its responses to agricultural practices in Australian agro-ecosystems: a review and synthesis. *Geoderma* 155 211–223. 10.1016/j.geoderma.2009.12.012

[B37] Luo Z. Wang E. Sun O. J. Smith C. J. Probert M. E. (2011). Modeling long-term soil carbon dynamics and sequestration potential in semi-arid agro-ecosystems. *Agric. For. Meteorol.* 151 1529–1544. 10.1016/j.agrformet.2011.06.011

[B38] Martin M. P. Wattenbach M. Smith P. Meersmans J. Jolivet C. Boulonne L. (2011). Spatial distribution of soil organic carbon stocks in France. *Biogeosciences* 8 1053–1065. 10.5194/bg-8-1053-2011

[B39] Mason M. (1992). Effect of management of previous cereal stubble on nitrogen fertiliser requirement of wheat. *Aust. J. Exp. Agric.* 32 355–362. 10.1071/EA9920355

[B40] Pankhurst C. E. McDonald H. J. Hawke B. G. Kirkby C. A. (2002). Effect of tillage and stubble management on chemical and microbiological properties and the development of suppression towards cereal root disease in soils from two sites in NSW. *Australia. Soil Biol. Biochem.* 34 833–840. 10.1016/S0038-0717(02)00014-7

[B41] Peverill K. I. Sparrow L. A. Reuter D. J. (1999). *Soil Analysis: An Interpretation Manual.* Collingwood: CSIRO Publishing.

[B42] Probert M. E. Dimes J. Keating B. Dalal R. Strong W. Strongb W. M. (1998). APSIM’s water and nitrogen modules and simulation of the dynamics of water and nitrogen in fallow systems. *Agric. Syst.* 56 1–28. 10.1016/S0308-521X(97)00028-0

[B43] Pricewaterhouse Coopers (2011). *The Australian Grains Industry - The Basics.* New York City, NY: Pricewaterhouse Coopers.

[B44] Queensland Government - Department of Science Information Technology Innovation and the Arts (2014). *SILO Climate Data [WWW Document].* Available at: http://www.longpaddock.qld.gov.au/silo/ [accessed on August 25, 2014].

[B45] Robertson F. Armstrong R. Partington D. Perris R. Oliver I. Aumann C. (2015). *Effect of Cropping Practices on Soil Organic Carbon: Evidence from Long-Term Field Experiments.* (Victoria: CSIRO Publishing) 53 636–646.

[B46] Sá J. C. M. Cerri C. C. Dick W. A. Lal R. Vesnke-Filho S. P. Piccolo M. C. (2001). Organic matter dynamics and carbon sequestration rates for a tillage chronosequence in a Brazilian Oxisol. *Soil Sci. Soc. Am. J.* 65 1486–1489. 10.2136/sssaj2001.6551486x

[B47] Sanderman J. Farquharson R. Baldock J. (2010). *Soil Carbon Sequestration Potential: A Review for Australian Agriculture.* Canberra: CSIRO Report.

[B48] Smettem K. R. J. Rovira A. D. Wace S. A. Wilson B. R. Simon A. Division C. (1992). Effect of tillage and crop rotation on the surface stability and chemical properties of a red-brown earth (Alfisol) under wheat. *Soil Tillage Res.* 22 27–40. 10.1016/0167-1987(92)90020-C

[B49] Smith P. Powlson D. S. Glendining M. J. Smith J. O. U. (1998). Preliminary estimates of the potential for carbon mitigation in European soils through no-till farming. *Glob. Chang. Biol.* 4 679–685. 10.1046/j.1365-2486.1998.00185.x

[B50] Smith P. Powlson D. S. Smith J. U. Falloon P. Coleman K. (2000). Meeting Europe’s climate change commitments: quantitative estimates of the potential for carbon mitigation by agriculture. *Glob. Chang. Biol.* 6 525–539. 10.1046/j.1365-2486.2000.00331.x

[B51] Soil Quality Pty Ltd (2014). *Soil Quality [WWW Document].* Available at: http://soilquality.org.au/ [accessed on August 25, 2014].

[B52] Sperow M. Eve M. Paustian K. (2003). Potential soil C sequestration on U.S. agricultural soils. *Clim. Change* 57 319–339. 10.1023/A:1022888832630

[B53] Standley J. Hunter H. M. Thomas G. A. Blight G. W. Webb A. A. (1990). Tillage and crop residue management affect Vertisol properties and grain sorghum growth over seven years in the semi-arid sub-tropics. 2. Changes in soil properties. *Soil Tillage Res.* 18 367–388. 10.1016/0167-1987(90)90121-S

[B54] The Soil Carbon Research Program - CSIRO Australian Universities and State Government Agencies (2014). *The Soil Carbon Research Program [WWW Document].* Available at: http://www.csiro.au/Organisation-Structure/Flagships/Sustainable-Agriculture-Flagship/Soil-Carbon-Research-Program/SCaRP-Projects-Overview.aspx [accessed on August 25, 2014].

[B55] Thorburn P. J. Probert M. E. Robertson F. A. (2001). Modelling decomposition of sugarcane surface residues with APSIM-Residue. *F. Crop. Res.* 70 223–232. 10.1016/S0378-4290(01)00141-1

[B56] Thorburn P. J. Robertson M. J. Clothier B. E. Snow V. O. Charmley E. Sanderman J. (2013). “Australia and New Zealand Perspectives on Climate Change and Agriculture,” in *ICP Series on Climate Change Impacts, Adaptation, and Mitigation — Vol. 2 HANDBOOK OF CLIMATE CHANGE AND AGROECOSYSTEMS Global and Regional Aspects and Implications, ICP Series on Climate Change Impacts, Adaptation, and Mitigation* ed. Lesmana M. (London: Imperial College Press) 320 10.1142/p755

[B57] Thorburn P. J. J. Biggs J. S. S. Collins K. Probert M. E. E. (2010). Using the APSIM model to estimate nitrous oxide emissions from diverse Australian sugarcane production systems. *Agric. Ecosyst. Environ.* 136 343–350. 10.1016/j.agee.2009.12.014

[B58] Unkovich M. Baldock J. Forbes M. (2010). *Variability in Harvest Index of Grain Crops and Potential Significance for Carbon Accounting: Examples from Australian Agriculture, Advances in Agronomy* 1st Edn Amsterdam: Elsevier Inc. 10.1016/S0065-2113(10)05005-4

[B59] Valzano F. P. Greene R. S. B. Murphy B. W. Rengasamy P. Jarwal S. D. (2001). Effects of gypsum and stubble retention on the chemical and physical properties of a sodic grey Vertosol in western Victoria F. *Aust. J. Soil Res.* 39 1333–1347. 10.1071/SR00045

[B60] Viscarra Rossel R. A. Webster R. Bui E. N. Baldock J. A. (2014). Baseline map of organic carbon in Australian soil to support national carbon accounting and monitoring under climate change. *Glob. Chang. Biol.* 20 2953–2970. 10.1111/gcb.12569 24599716 PMC4258068

[B61] White P. (1990). The influence of alternative tillage systems on the distribution of nutrients and organic-carbon in some common Western Australian wheatbelt soils. *Aust. J. Soil Res.* 28 95–116. 10.1071/SR9900095

[B62] Yan H. Cao M. Liu J. Tao B. (2007). Potential and sustainability for carbon sequestration with improved soil management in agricultural soils of China. *Agric. Ecosyst. Environ.* 121 325–335. 10.1016/j.agee.2006.11.008

[B63] Yang X. M. Kay B. D. (2001). Impacts of tillage practices on total, loose- and occluded-particulate, and humified organic carbon fractions in soils within a field in southern Ontario. *Can. J. Soil Sci.* 81 149–156. 10.4141/S00-015

[B64] Zhao G. Bryan B. A. King D. Luo Z. Wang E. Song X. (2013). Impact of agricultural management practices on soil organic carbon: simulation of Australian wheat systems. *Glob. Chang. Biol.* 19 1585–1597. 10.1111/gcb.12145 23504769

